# Overfeeding in the early postnatal period aggravates inflammation and hepatic insulin sensitivity in the 5α-dihydrotestosterone-induced animal model of PCOS

**DOI:** 10.3389/fendo.2024.1402905

**Published:** 2024-08-29

**Authors:** Nataša Veličković, Bojana Mićić, Ana Teofilović, Milena Milovanovic, Mirna Jovanović, Ana Djordjevic, Djuro Macut, Danijela Vojnović Milutinović

**Affiliations:** ^1^ Department of Biochemistry, Institute for Biological Research “Siniša Stanković” - National Institute of the Republic of Serbia, University of Belgrade, Belgrade, Serbia; ^2^ Université Paris Cité, Learning Planet Institute (LPI), Paris, France; ^3^ Clinic for Endocrinology, Diabetes and Metabolic Diseases University Clinical Centre of Serbia, Faculty of Medicine, University of Belgrade, Belgrade, Serbia

**Keywords:** polycystic ovary syndrome, postnatal overfeeding, insulin resistance, obesity, inflammation

## Abstract

**Background:**

Polycystic ovary syndrome (PCOS) is the most common endocrine disorder in women of reproductive age and is closely associated with chronic low-grade inflammation and insulin resistance. To clarify the contribution of prepubertal weight gain to the development of insulin resistance in PCOS, we investigated the effects of early postnatal overfeeding on inflammatory and energy-sensing pathways as well as on markers of insulin signaling in the liver of the PCOS rat model.

**Methods:**

Obesity induced by overfeeding was achieved by reducing litter size, while the PCOS-like condition was developed by treatment with 5α-dihydrotestosterone (DHT). Western blot and qPCR were used to analyze the expression of pro-inflammatory transcription factors and cytokines, as well as markers of the energy sensing and insulin signaling pathways.

**Results:**

The results showed that hepatic insulin sensitivity was impaired only in DHT-treated rats raised in small litters, as evidenced by increased phosphorylation of IRS1 on Ser307 and decreased expression of total IRS1. Postnatal overfeeding stimulated JNK1 activation independent of hyperandrogenemia; nevertheless, the synergistic effect of both factors triggered NLRP3 activation and increased IL1β expression in the small litter DHT-treated group. This pro-inflammatory state was accompanied by decreased activatory phosphorylation of AMPK and reduced levels of its protein targets.

**Conclusions:**

Overfeeding in the early postnatal period leads to a decrease in hepatic insulin sensitivity in the rat model of PCOS, which is associated with decreased activation of AMPK and stimulation of the hepatic NLRP3-IL1β signaling pathway. Accordingly, the inhibition of NLRP3 activation could provide a basis for the development of new therapeutic strategies for the treatment of insulin resistance in women with PCOS.

## Introduction

Polycystic ovary syndrome (PCOS) is one of the most common reproductive disorders with a variety of endocrine symptoms and is often associated with infertility in women of reproductive age (1). The prevalence of PCOS is estimated to be between 6% (using earlier, more restrictive diagnostic criteria) and 20% (using current, more comprehensive definitions) (2). PCOS is often associated with obesity, abdominal adiposity, metabolic disorders and increased cardiovascular risk.

The visceral type of obesity in particular is characteristic of PCOS patients, and according to Lim et al. the average prevalence of obesity in women with PCOS is around 49% (3). In addition, it is known that the severity of PCOS features usually worsens when it is accompanied by obesity (4). Obesity is a metabolic condition that is closely associated with chronic, low-grade inflammation characterized by higher levels of pro-inflammatory cytokines, chemokines and oxidative stress markers. Furthermore, these inflammatory processes may represent a link between PCOS and impaired insulin signaling and glucose metabolism at local and systemic levels (4, 5).

Adipose tissue inflammation and dysfunction lead to release of inflammatory mediators, adipokines and fatty acids to circulation, resulting in increased lipid flux into the liver and activation of hepatic proinflammatory signaling pathways (6). The main regulators of hepatic metabolic inflammation are NFκB and JNK (7) that can be triggered by many mediators, including increased intrahepatic saturated fatty acids (SFA), gut-derived lipopolysaccharide (LPS) and lipotoxic products. They further lead to the stimulation of toll-like receptor 4 (TLR4), which in turn leads to the activation of NFκB and JNK1 and the subsequent production of inflammatory cytokines and mediators (8). Subsequently, NFκB and JNK1 lead to the activation of the NOD-like receptor protein 3 (NLRP3) inflammasome and caspase 1-dependent release of the pro-inflammatory cytokines IL1β and IL18 (9). The NLRP3 inflammasome is associated with various high-risk reproductive disorders and is therefore considered a novel therapeutic target for PCOS (10). In addition, NLRP3 interacts with protein kinase Cϵ (PKCϵ) in hepatocytes, leading to the development of hepatic insulin resistance (11).

AMPK, an important energy sensor, is located at the interface between inflammation and insulin resistance, where it balances energy homeostasis in response to energy sources and demands (12). Reduced activity of AMPK induced by low-grade chronic inflammation has been implicated in several diseases, including type 2 diabetes and atherosclerosis (13). Dysregulation of AMPK is correlated with both endocrine and reproductive function, and activation of AMPK by metformin is considered the first therapeutic approach in PCOS patients (14).

In this study we used a well-established DHT-induced animal model of PCOS that was additionally challenged by postnatal overfeeding (15). Early postnatal overfeeding in this PCOS model, induced by litter size reduction, resulted in animal weight gain and visceral adiposity, mimicking prepubertal obesity (16), which could be a possible trigger for the metabolic features of PCOS (17). Previously, we have shown that this animal model develops both reproductive and metabolic features of the syndrome, including decreased systemic insulin sensitivity and hyperinsulinemia (18). Our hypothesis was that the insulin resistance associated with PCOS is related to hepatic inflammation on the one hand, and a dysregulated AMPK signaling pathway on the other. To test this hypothesis, we analyzed inflammatory and energy-sensing pathways as well as the insulin signaling pathway in the liver of DHT-treated animals exposed to postnatal overfeeding.

## Material and methods

### Study design and animals

The study was conducted on female Wistar rats that were subjected to two consecutive treatments. On the second postnatal day, in addition to litters of average size (normal litters, NL) consisting of 10 pups with lactating dams, small litters (SL), consisting of three pups with nursing mothers, were formed to introduce the factor of early postnatal overfeeding. Upon weaning, on postnatal day 22, continuous treatment with 5α-dihydrotestosterone (DHT) was introduced to stimulate hyperandrogenemia. Hormonal pellets containing 7.5 mg of DHT, which was continuously released at a daily dose of 83 µg during the 90-day treatment, or placebo pellets without active substance (DHT or placebo) were implanted subcutaneously into the neck region of the animals according to the manufacturer’s instructions (Innovative Research of America, USA). The dose chosen corresponded to the androgen levels in the blood of women with PCOS (19). Four experimental groups were formed in this way: NL-placebo, NL-DHT, SL-placebo and SL-DHT.

After weaning and implantation of the pellets, all animals were housed three per cage under standard conditions and had *ad libitum* access to standard laboratory chow and tap water. At the end of the hormonal treatment, the animals were sacrificed by rapid decapitation in the diestrus phase of the estrus cycle after 6 hours of fasting. Trunk blood was collected for biochemical analyses, while livers, subcutaneous and visceral adipose tissues (SAT and VAT, respectively) were carefully removed, measured and frozen in liquid nitrogen for further analyses. Adiposity index was calculated as sum of SAT and VAT masses divided by the body mass and multiplied by 100 (%).

All procedures were performed in accordance with Directive 2010/63/EU on the protection of animals used for experimental and other scientific purposes. The study was approved by the Ethical Committee for the Use of Laboratory Animals of the Institute for Biological Research “Siniša Stanković” of the University of Belgrade (No. 01-02/19).

### Protein sample preparation and Western blot

Whole cell extracts as well as nuclear and cytoplasmic protein fractions were prepared for Western blot analyses. Whole cell extracts were prepared in ice-cold RIPA buffer as described in Teofilović et al. (20), while for the preparation of cytoplasmic and nuclear protein fractions frozen livers were homogenized in ice-cold homogenization buffer (20 mM Tris HCl pH 7.2, 10% glycerol, 50 mM NaCl, 1 mM EDTA Na_2_, 1 mM EGTA Na_2_, 2 mM DTT, protease and phosphatase inhibitors) and further processed as previously described in Mićić et al. (18). Protein concentration in all samples was determined by the method described by Spector (21), using bovine serum albumin as a standard.

Samples were boiled for 5 min in 2 × Laemmli buffer, and 40 µg of proteins were run together with a pre-stained protein ladder (10–170 kDa, Thermo Fisher Scientific, Waltham, MA, USA) on sodium dodecyl sulphate-polyacrylamide gels using the Mini-Protean II Electrophoresis Cell system (Bio-Rad Laboratories, Hercules, CA, USA). The proteins were transferred to polyvinylidene difluoride (PVDF) membranes (Immobilon-FL, Millipore, USA) in transfer buffer (25 mM Tris, 192 mM glycine, 20% methanol v/v), at 135 mA, 4°C, overnight, in the Mini Trans-Blot Electrophoretic Transfer Cell system (Bio-Rad Laboratories, USA). Membranes were blocked with 5% BSA or nonfat milk for 90 minutes at room temperature and probed overnight at 4°C with the following primary antibodies: anti-pIRS1^Ser307^ (ab5599, 1:500) purchased from Abcam (UK); anti-pJNK^Thr183/Tyr185^ (sc-6254, 1:500), anti-JNK (sc-571, 1:500), anti-NFκB (sc-8008, 1:500), anti-SIRT1 (sc-15404, 1:500), anti-AMPKα1/2 (sc-25792, 1:1000), purchased from Santa Cruz Biotechnology (USA); anti-IRS1 (2382s, 1:1000), anti-pAMPKα1/2^Thr172^ (2535s, 1:1000), anti-pACC^Ser79^ (3661s, 1:1000) purchased from Cell Signaling (USA), and anti-NLRP3 (NBP2-1024-46, 1:1000) purchased from Novus Biologicals (USA). To confirm equal protein loading, β-actin (ab8227, Abcam, UK; 1:10000), and lamin B1 (sc-374015, Santa Cruz Biotechnology, USA; 1:500) were used. Membranes were washed extensively in PBS containing 0.1% Tween-20 and incubated for 90 minutes with the appropriate mouse (ab97046, Abcam, UK; 1:30000) or rabbit (ab6721, Abcam, UK; 1:20000) HRP-conjugated secondary antibodies. The immunoreactive bands were visualized by the enhanced chemiluminescence method using the iBright FL1500 Imaging System, and quantitative analysis was performed using iBright Analysis Software (Thermo Fisher Scientific, USA).

### RNA isolation, cDNA preparation and real-time PCR

Total RNA was extracted from liver with TRIzol reagent (Invitrogen, USA) according to the manufacturer’s instructions. The concentration and purity of the isolated RNA was determined spectrophotometrically at 260 nm (Nano Photometer N60, Implen GmbH, Germany), whereby an absorbance ratio 260/280 of more than 1.8 is considered satisfactory. Complementary DNA (cDNA) was prepared from 2 μg of total RNA using the High Capacity Reverse Transcription Kit (Applied Biosystems, USA) according to the manufacturer’s instructions and used for real time PCR reactions.

The expression of genes encoding TLR4, TNFα, IL1β, IL6 was quantified by SYBR® Green qPCR, with hypoxanthine guanine phosphoribosyl transferase (HPRT) as a reference gene. The following primer sets were used: *Tlr4* (F: ATC ATC CAG GAA GGC TTC CA, R: GCT AAG AAG GCG ATA CAA TTC), *Hprt* (F: CAG TCC CAG CGT GAT TA, R: AGC AAG TCT TTC AGT CCT GTC) purchased from Invitrogen, USA; *Il1β* (F: AGC AGC TTT CGA CAG TGA GG, R: CTC CAC GGG CAA GAC ATA GG), *Il6* (F: GTT TCT CTC CGC AAG AGA CTT, R: ATA CTG GTC TGT TGT GGG TGG), *Tnfα* (F: GCC ACC CTG TTC TGT CT, R: CGC TTG GTG GTT TGC TAC GAC) acquired from Microsinth, Switzerland. The reaction mix contained 1 × power SYBR® Green PCR master mix, specific primer sets, and cDNA template. Thermal cycling conditions were: 2 min incubation at 50°C, 10 min at 95°C, followed by 40 cycles at 95°C for 15 s and 60°C for 60 s. To confirm the formation of a single PCR product, melting curve analyses were performed at the end of each experiment. To exclude possible reagent contamination, no template controls were used for each target gene. TaqMan gene expression FAM labeled probe (Applied Biosystems Assay-on-Demand Gene Expression Products, USA) was used to determine the expression of gene for macrophage migration inhibitory factor (MIF) (*Mif*, Rn00821234_g1*), where TATA-box protein (TBP) (Rn01455646_m1*) was used as internal control for quantitative normalization of cDNA (22). The TaqMan reaction mix consisted of 1 × TaqMan® universal PCR master mix, with AmpErase UNG, 1 × TaqMan® gene expression assay, and cDNA template, while thermal cycling conditions were the same as for SYBR® Green reactions. Relative gene expression was calculated using the comparative 2^-ΔΔCt^ method (23).

### Statistical analyses

All data are presented as mean ± standard error of the mean (SEM). Two-way repeated measures ANOVA followed by Sidak’s *post hoc* test was used to compare masses of animals throughout life. The effects of the factors examined (litter size reduction and DHT treatment) and the interaction between them were determined by a two-way ANOVA. Tukey’s *post hoc* test was applied to assess the differences between the groups. Statistical significance was assumed at p < 0.05. Analyses were performed with GraphPad Prism 8 (San Diego, USA) and STATISTICA 7.0 software package (StatSoft Inc., Tulsa, USA).

## Results

### Litter size reduction increases weight gain and adiposity index, while DHT treatment in combination with litter size reduction affect relative liver mass

**Figure 1:**
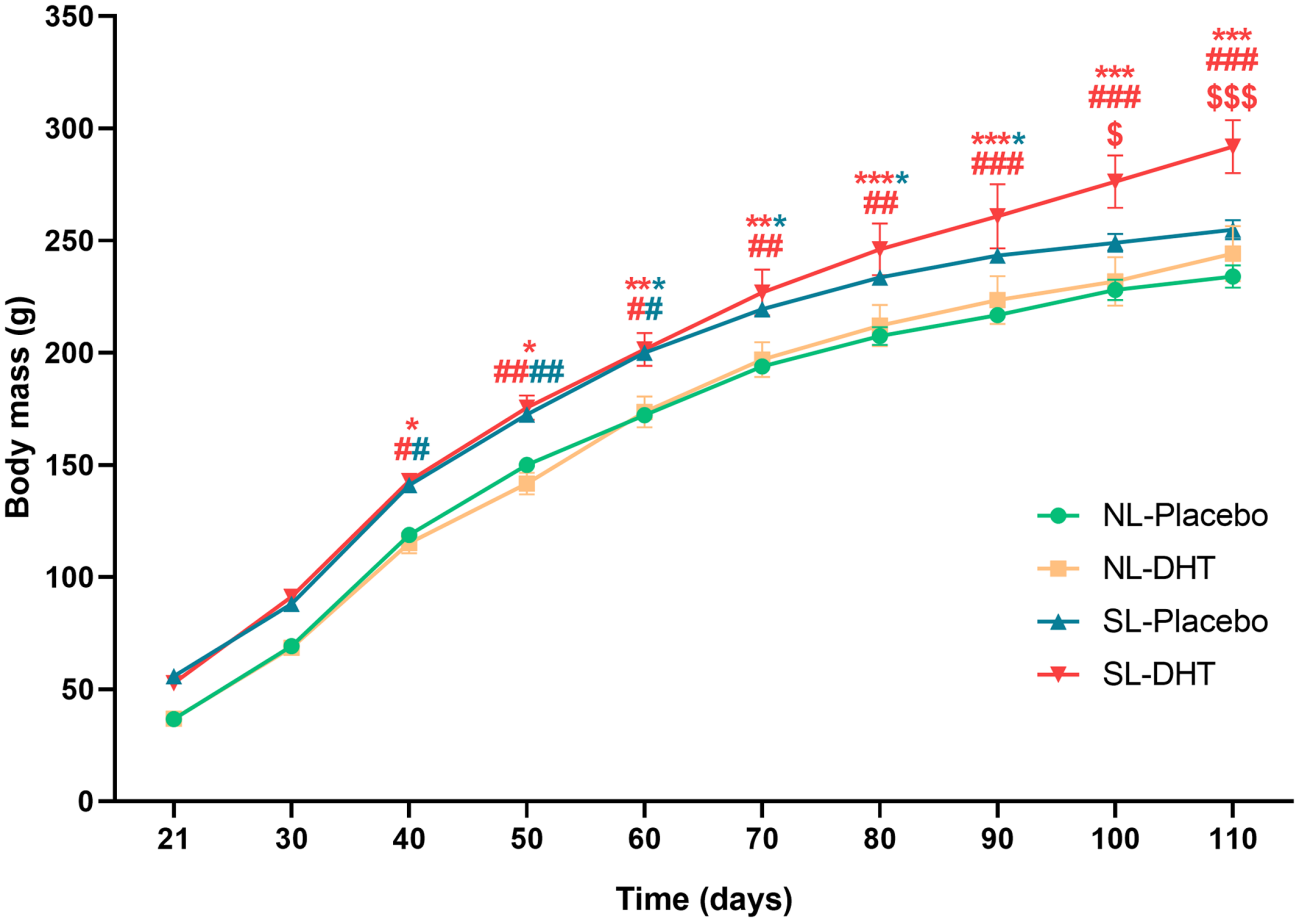
The effects of litter size reduction and DHT treatment on weight gain after weaning period. Each point represents the mean ± SEM (n = 6). Comparison between body masses of animals from small and normal litters by time was performed by two-way repeated measures ANOVA followed by Sidak’s *post hoc* test. Different symbols denote the values that are significantly different from NL-Placebo group (^*^p < 0.05, ^**^p < 0.01, ^***^p < 0.001), NL-DHT group (^#^p < 0.05, ^##^p < 0.01, ^###^p < 0.001) and SL-Placebo group (^$^p < 0.05, ^$$$^p < 0.001).

Animals raised in small litters during weaning period gained significantly more weight before the beginning of hormonal treatment in comparison to animals from normal litters (F_1, 20_ = 243.33, p < 0.001) (**Figure 1**). Throughout the whole experiment weight gain was equal in normal litters regardless of DHT treatment, while animals from small litter treated with DHT had more prominent weight gain as compared with postnatally overfed placebos. At the end of the experiment, both litter size (F_1, 20_ = 12.35, p < 0.01) and DHT (F_1, 20_ = 5.71, p < 0.05) had effect on body mass, which was higher in DHT treated animals raised in small litter in comparison to all others groups (SL-DHT *vs*. NL-Placebo, NL-DHT, SL-Placebo, p < 0.001) (**Figure 1**). However, when comparing the weight gain curves, litter size was the main factor promoting the increase in body mass (F_1, 20_ = 26.34, p < 0.001; SL-DHT *vs*. NL-Placebo, ^**^p < 0.01; SL-DHT *vs*. NL-DHT; ^##^p < 0.01, SL-Placebo *vs.* NL-Placebo; ^*^p < 0.05, SL-Placebo *vs*. NL-DHT, ^#^p < 0.05) (**Table 1**). This could be the result of an early programmed increase in food intake in small litter DHT-treated animals, as there is less competition for available food in the small litters (SL-DHT *vs*. NL-Placebo, NL-DHT, SL-Placebo, p < 0.05). Litter size was also the factor that increased adiposity index (F_1, 20_ = 13.97, p < 0.01; SL-Placebo *vs*. NL-Placebo, ^*^p < 0.05; SL-Placebo *vs.* NL-DHT, ^#^p < 0.05) (**Table 1**). Both factors had effect on relative liver mass (litter size: F_1, 20_ = 13.50, p < 0.01; DHT: F_1, 20_ = 8.77, p < 0.01) that was decreased in animals from small litters due to increase in their body mass (SL-DHT *vs*. NL-Placebo, ^***^p < 0.001; SL-Placebo *vs.* NL-Placebo, ^*^p < 0.05) (**Table 1**).

**Table 1:** The effects of litter size reduction and DHT treatment on food intake and morphometrical parameters.

	NL-Placebo	NL-DHT	SL-Placebo	SL-DHT	Two-way ANOVA		
					Litter size	DHT	Litter size x DHT
**Food intake (g/day/cage)**	44 ± 0.60	41 ± 0.50	42 ± 0.50	51 ± 2.2^*#$^	<0.05	NS	<0.01
**Weight gain AUC** **(g x days)**	11603 ± 255	11726 ± 581	13687 ± 196^*#^	14592 ± 699^**##^	<0.001	NS	NS
**Adiposity index (%)**	4.36 ± 0.52	4.06 ± 0.51	6.23 ± 0.54^*#^	5.24 ± 0.39	<0.01	NS	NS
**Mass of liver (g)**	7.85 ± 0.24	7.60 ± 0.53	7.78 ± 0.28	7.88 ± 0.58	NS	NS	NS
**Liver to body mass ratio (x100)**	3.35 ± 0.05	3.10 ± 0.08	3.05 ± 0.09^*^	2.88 ± 0.06^***^	<0.01	<0.01	NS

Area under the curve (AUC) of weight gain, adiposity index, and absolute and relative liver mass were measured and calculated in female Wistar rats raised in normal or small litters and treated with DHT or placebo pellets. All data are presented as means ± SEM (n = 6). Two-way ANOVA followed by Tukey’s post hoc test was performed to determine the effects of litter size and DHT treatment, as well as their interaction. Different symbols denote significant differences from NL-Placebo group (*p < 0.05, **p <0.01, ***p < 0.001), NL-DHT group (**^#^** p < 0.05, ^##^p < 0.01) and SL-Placebo group (^$^p < 0.05). Bold values are used to be easier to notice the differences with statistical significance.

### DHT treatment in combination with litter size reduction impair hepatic insulin sensitivity

**Figure 2:**
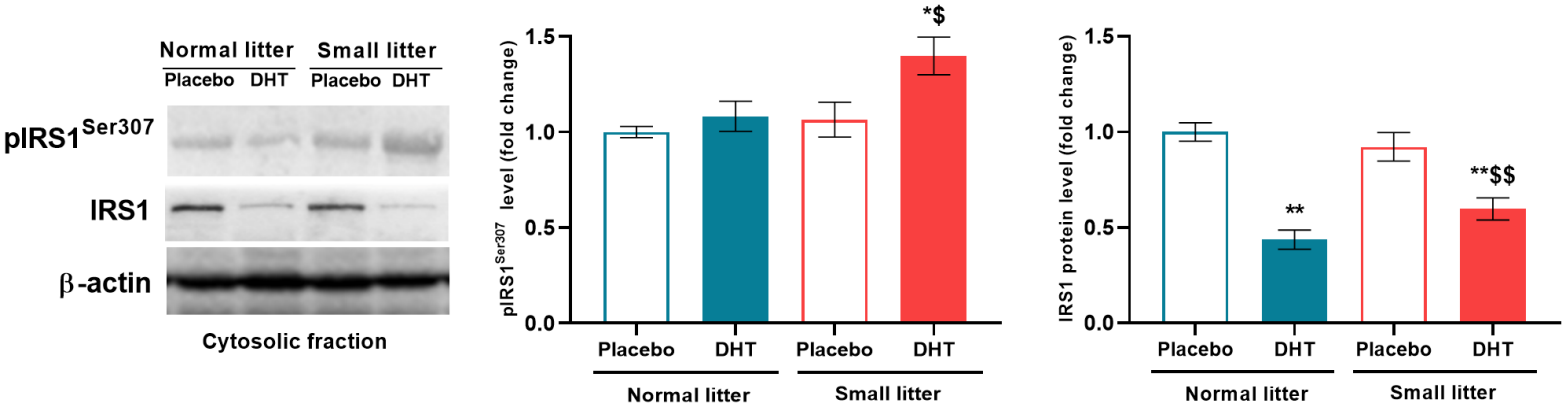
The levels of phosphorylated and total IRS1 protein in the liver after postnatal overfeeding and DHT treatment. Representative Western blots and relative quantification of phospho-IRS1 (Ser^307^) and total IRS1 in the cytosolic fraction. β-actin was used as loading control. All data are presented as mean ± SEM (n = 6). Two-way ANOVA followed by Tukey’s *post hoc* test was performed to determine the effects of litter size reduction and DHT treatment, as well as their interaction. Different symbols denote significant differences from NL-Placebo (^*^p < 0.05, ^**^p < 0.01) and SL-Placebo group (^$^p < 0.05, ^$$^p < 0.01).

In order to analyze the impact of litter size reduction and DHT treatment on insulin signaling in the liver, the protein level of IRS1 and its inhibitory phosphorylation on Ser^307^ were estimated. Two-way ANOVA showed that both litter size (F_1, 18_ = 4.77, p < 0.05) and DHT treatment (F_1, 18_ = 5.68, p < 0.05) affected the level of IRS1 phosphorylation on Ser^307^, so that it was increased in small litter treated with DHT compared to both placebo groups (SL-DHT *vs*. NL-Placebo, SL-Placebo, p < 0.05) (**Figure 2**). As a result of DHT treatment (F_1, 20_ = 59.70, p < 0.001) and the interaction between factors (F_1, 20_ = 5.35, p < 0.05) total IRS1 protein was decreased in DHT-treated animals from normal and small litters in comparison to placebos from normal litter (NL-DHT *vs*. NL-Placebo, SL-DHT *vs*. NL-Placebo, **p < 0.01). Additionally, the level of total IRS1 in DHT-treated animals raised in small litter was decreased in comparison with controls from the same litters (SL-DHT *vs*. SL-Placebo, ^$$^p < 0.01) (**Figure 2**).

### Early postnatal overfeeding and DHT treatment affect inflammatory status of the liver

**Figure 3:**
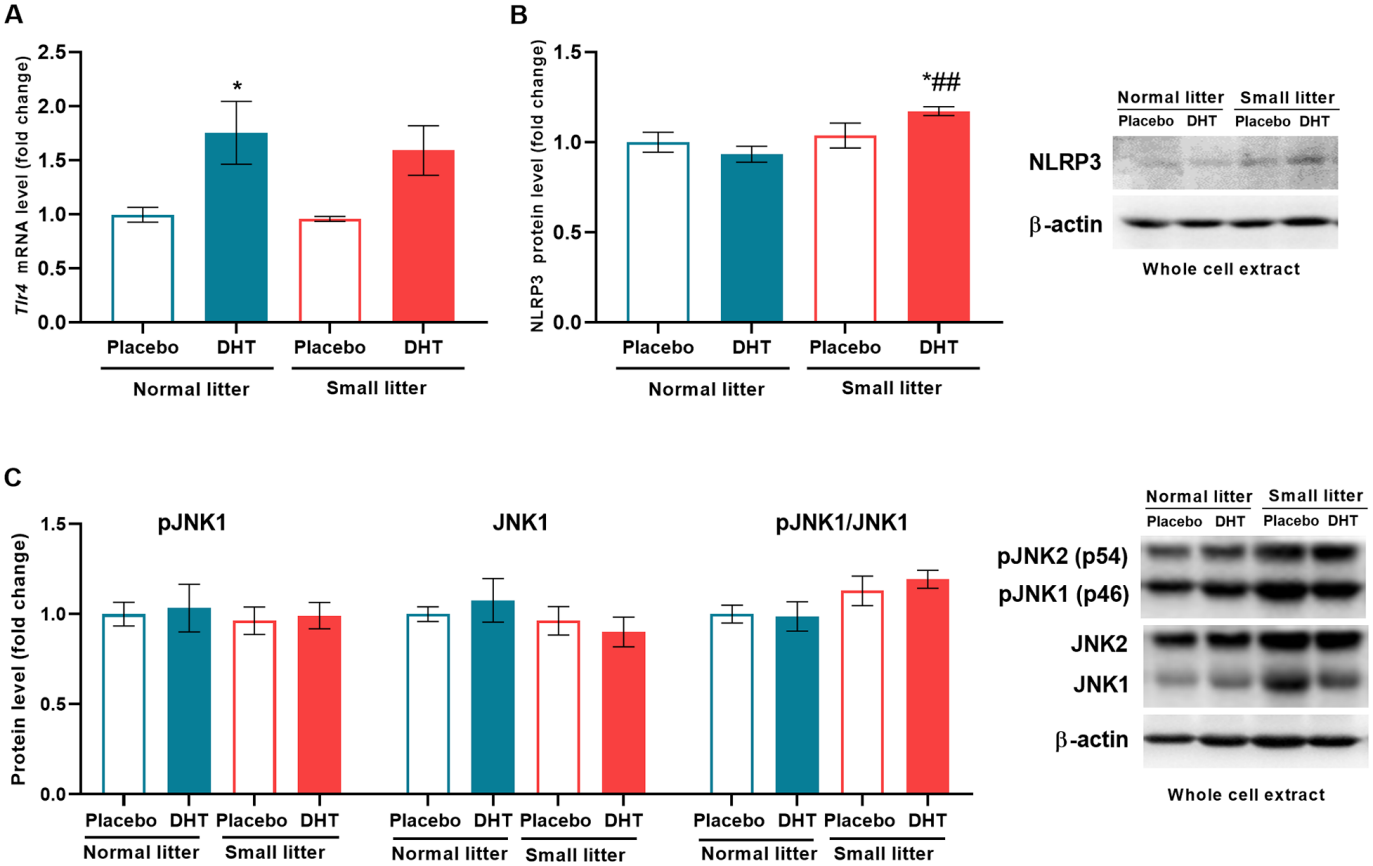
The effects of litter size and DHT treatment on inflammatory mediators in the liver. **(A)** Relative expression of *Tlr4* gene and **(B)** relative quantification with representative Western blots of NLRP3 protein and **(C)** phosphorylated (Thr183/Tyr185) and total form of JNK in the whole cell extract. Quantification of mRNA levels was done relative to the amount of *Hprt*, while β-actin was used as protein loading control. All data are presented as mean ± SEM (n = 6). Two-way ANOVA followed by Tukey’s *post hoc* test was performed to determine the effects of litter size reduction and DHT treatment, as well as their interaction. Different symbols denote significant differences from NL-Placebo group (^*^p < 0.05) and NL-DHT group (^##^p < 0.01).

The potential impact of factors from adipose tissue and the intestine, such as free fatty acids (FFA) and lipopolysaccharides, on inflammatory processes in the liver was determined by analyzing the expression of hepatic TLR4 gene (24). DHT treatment had main effect on *Tlr4* (F_1, 18_ = 16.31, p < 0.001) that led to its increase in DHT-treated group from normal litter in comparison to placebos (NL-DHT *vs*. NL-Placebo, ^*^p < 0.05) (**Figure 3A**). On the other hand, litter size alone (F_1, 19_ = 8.87, p < 0.001) as well as interaction of both factors (F_1, 19_ = 5.04, p < 0.05) influenced the protein level of NLRP3 inflammasome. The *post hoc* showed that NLRP3 was activated in DHT-treated group from small litter in comparison to placebo and DHT treated animals raised in normal litters (SL-DHT *vs*. NL-Placebo, ^*^p < 0.05; SL-DHT *vs*. NL-DHT, ^##^p < 0.01) (**Figure 3B**). The litter size also had effect on the ratio between activatory phosphorylation of JNK and its total protein form (F_1, 18_ = 5.47, p < 0.05) but without *post hoc* differences between the groups (**Figure 3C**).

**Figure 4:**
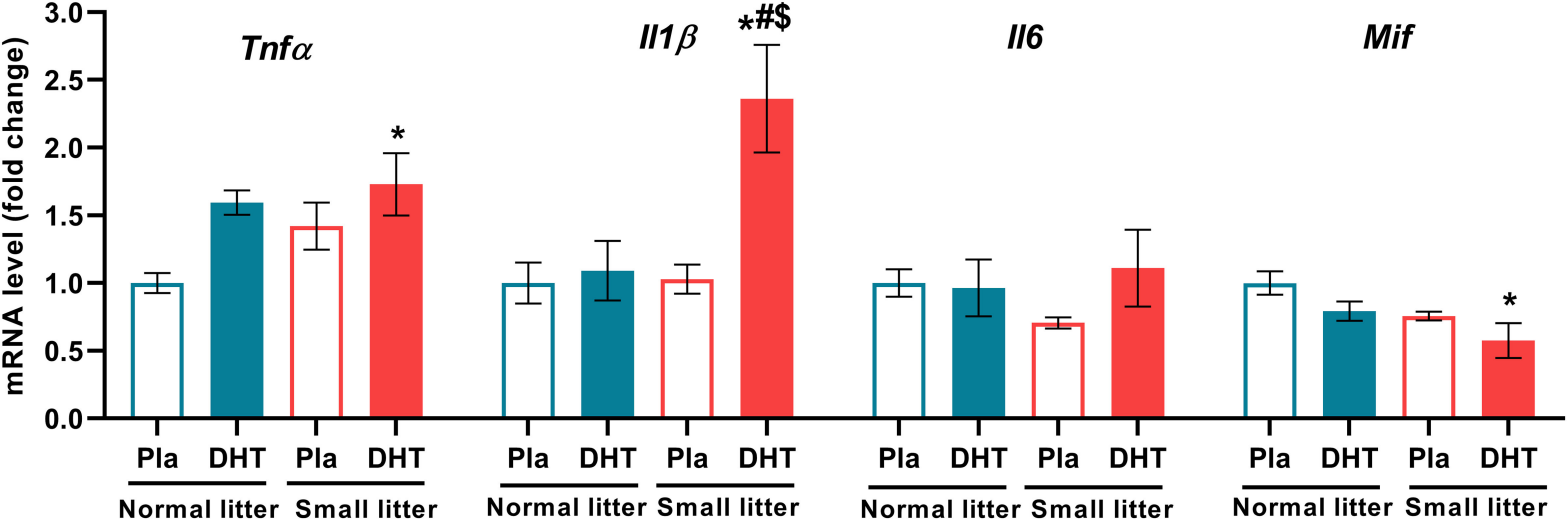
The levels of hepatic proinflammatory cytokines after postnatal overfeeding and DHT treatment. Relative expression of *Tnfα*, *Il1β*, *Il6* and *Mif* gene. Quantification of mRNA levels was done relative to the amount of *Hprt*. All data are presented as mean ± SEM (n = 6). Two-way ANOVA followed by Tukey’s *post hoc* test was performed to determine the effects of litter size reduction and DHT treatment, as well as their interaction. Different symbols denote significant differences from NL-Placebo group (*p < 0.05), NL-DHT group (^#^p < 0.05) and SL-Placebo group (^$^p < 0.05).

DHT treatment had main effect on the mRNA level of proinflammatory cytokine *Tnfα* (F_1, 18_ = 5.79, p < 0.05), while interaction of both factors affected *Il1β* (litter size x DHT: F_1, 20_ = 6.38, p < 0.05). Treatment with DHT (F_1, 19_ = 4.66, p < 0.05), as well as litter size reduction (F_1, 19_ = 6.54, p < 0.05) affected the mRNA level of *Mif*, while treatments did not have any effect on *Il6* level (**Figure 4**). These proinflammatory factors were changed only in DHT group of animals raised in small litter; *Tnfα* was increased in comparison to placebos raised in normal litter (SL-DHT *vs*. NL-Placebo, *p < 0.05), while *Il1β* was elevated in comparison to all groups (SL-DHT *vs*. NL-Placebo, NL-DHT, SL-DHT, p < 0.05). On the contrary, the level of *Mif* was decreased in DHT animals from small litter in regard to placebos from normal litter size (SL-DHT *vs*. NL-Placebo, ^*^p < 0.05) (**Figure 4**).

**Figure 5:**
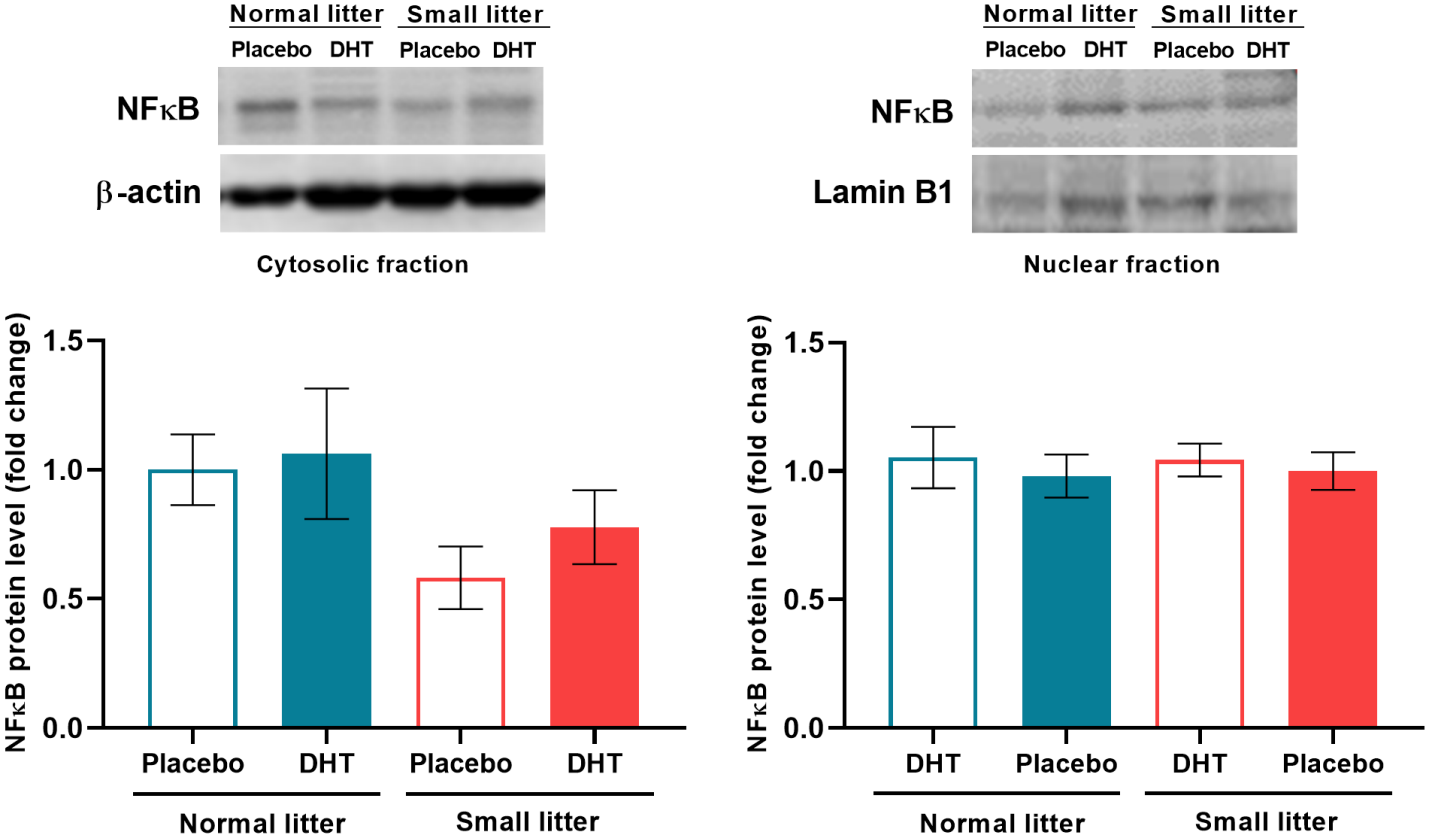
The effects of litter size and DHT treatment on the level and subcellular distribution of transcriptional regulator NFκB in the liver. Representative Western blots and relative quantification of NFκB protein in the cytosolic and nuclear fraction. β-actin and lamin B1 were used as loading controls for cytosolic and nuclear fraction, respectively. All data are presented as mean ± SEM (n = 6). Two-way ANOVA followed by Tukey’s *post hoc* test was performed to determine the effects of litter size reduction and DHT treatment, as well as their interaction.

The reduction in litter size only affected the protein content of the proinflammatory transcriptional regulator NFκB in the hepatic cytosolic fraction (F_1, 19_ = 4.48, p < 0.05), but with no effect on the differences between groups. However, the treatments did not alter NFκB levels in the nuclear fraction (**Figure 5**).

### Litter size reduction followed by DHT treatment decreases the level of energy sensors in the liver

**Figure 6:**
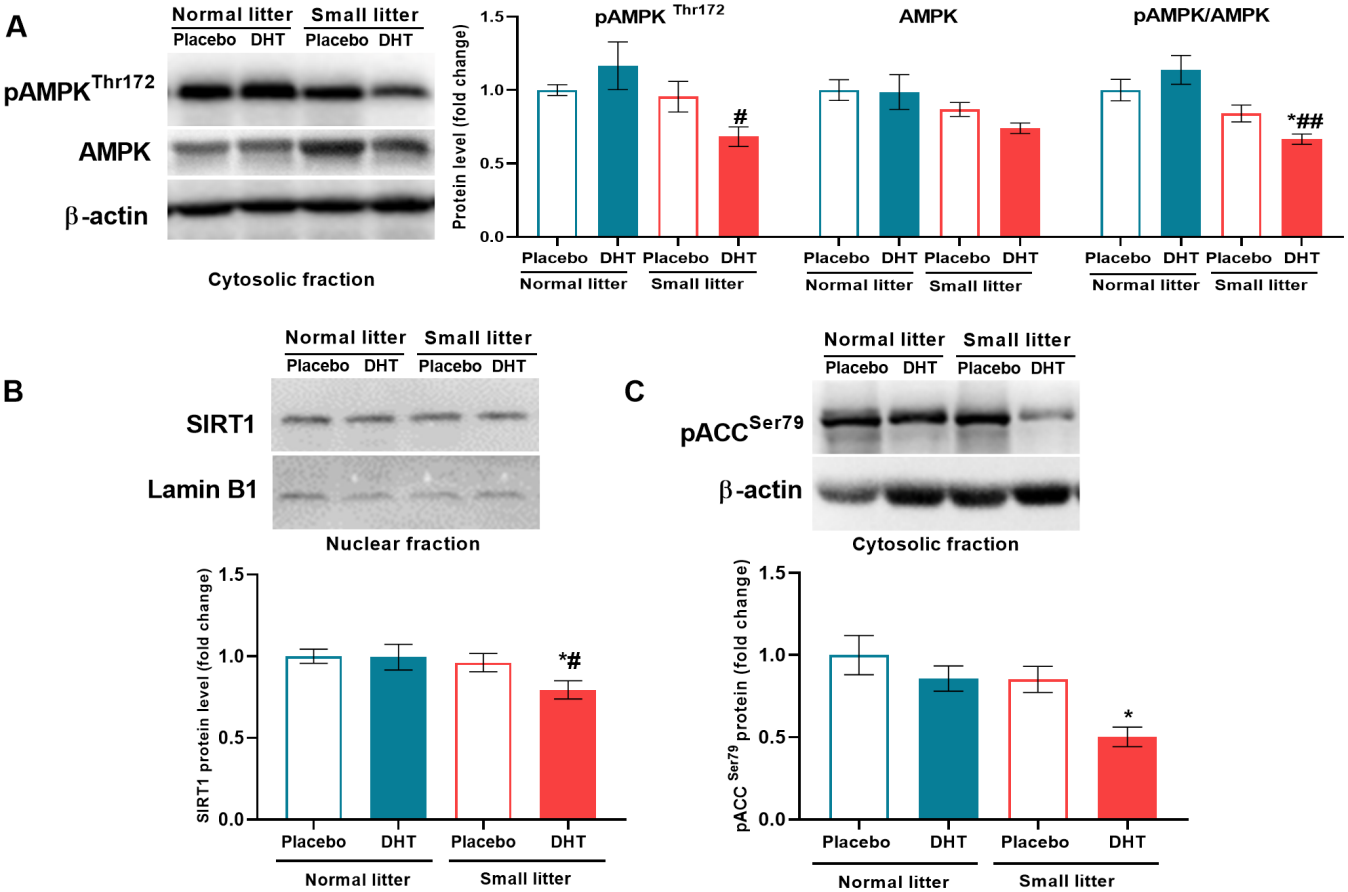
The level of energy sensors in the liver after postnatal overfeeding and DHT treatment. Representative Western blots and relative quantification of **(A)** phospho-AMPK (Thr^172^) and total AMPK protein in the cytosolic fraction, **(B)** SIRT1 protein in the nuclear fraction and **(C)** phospho-ACC (Ser^79^) in the cytosolic fraction. β-actin and lamin B1 were used as loading controls for cytosolic and nuclear fraction, respectively. All data are presented as mean ± SEM (n = 6). Two-way ANOVA followed by Tukey’s *post hoc* test was performed to determine the effects of litter size reduction and DHT treatment, as well as their interaction. Symbols denote significant differences from NL-Placebo group (^*^p < 0.05) and NL-DHT group (^#^p < 0.05, ^##^p < 0.01).

Litter size was the main factor affecting the activatory phosphorylation of AMPK on Thr^172^ (F_1, 19_ = 5.90, p < 0.05) and its total protein level (F_1, 20_ = 6.30, p < 0.05) (**Figure 6A**), while the ratio between phospho and total AMPK was also influenced by both litter size (F_1, 18_ = 18.31, p < 0.001) and interaction of factors (F_1, 18_ = 4.59, p < 0.05). *Post hoc* analyses revealed that the level of phospho-AMPK was decreased in the DHT treated animals from small litter in comparison to DHT treated animals raised in normal litter (SL-DHT *vs*. NL-DHT, ^#^p < 0.05), while the ratio phospho/total AMPK was decreased in DHT-treated animals raised in small litter in comparison to both normal litter groups (SL-DHT *vs.* NL-Placebo, NL-DHT, ^*^p < 0.05, ^##^p < 0.01, respectively). SIRT1 protein level was affected by the litter size reduction (F_1, 19 =_ 5.95, p < 0.05) and *post hoc* analysis showed that its level was decreased in the SL-DHT group in comparison to NL-Placebo and NL-DHT (^*^p < 0.05, ^#^p < 0.05, respectively) (**Figure 6B**). In order to confirm the impact of applied treatments on AMPK activation, its targeted phosphorylation on Ser^79^ of ACC enzyme was determined. Both litter size (F_1, 20_ = 5.52, p < 0.05) and DHT treatment (F_1, 20_ = 5.26, p < 0.05) affected phospho-ACC, and its level was decreased in DHT treated animals from small litter in comparison to placebo group raised in normal litter (SL-DHT *vs*. NL-Placebo, ^*^p < 0.05) (**Figure 6C**).

## Discussion

In this study we used an established DHT-induced animal model of PCOS that was additionally exposed to postnatal overfeeding, which mimics the obese phenotype in adolescents and increases the likelihood of PCOS occurrence (17). We have previously shown that the animals in this model develop both reproductive and metabolic features of the syndrome, including weight gain, increased visceral adiposity, hyperinsulinemia, and decreased systemic insulin sensitivity (25). It is noteworthy that the individual treatments in our previous study had no effect on the insulin sensitivity of the animals (15), but that the interaction of both treatments, hyperandrogenemia and early postnatal overfeeding, was necessary for the development of hyperinsulinemia and reduced insulin sensitivity, as determined by the intraperitoneal glucose tolerance test (18). The cause of the reduced glucose tolerance observed in the small litter DHT-treated animals may be due to impaired insulin signaling in muscle, adipose tissue and liver. We have previously demonstrated defects at critical nodes of the insulin signaling pathway in skeletal muscle, associated with reduced glucose uptake (25), which was not associated with altered insulin signaling in the visceral adipose tissue (18). One of the hallmarks of insulin resistance is increased phosphorylation of IRS1 protein at Ser307, which inhibits the insulin receptor signaling cascade (26). In this study, we detected increased IRS1^Ser307^ phosphorylation in the liver tissue of the small litter raised DHT-treated animals, suggesting impaired hepatic insulin signaling. This is consistent with a previous study, in which increased IRS1^Ser307^ phosphorylation was observed in the liver of PCOS patients, along with decreased expression of pIRS1^Tyr1150/1151^, pAkt^Thr308^ and pERK1/2^Thr202/Tyr204^ (27). In addition to increased phosphorylation at Ser307 in the small litter DHT-treated group, we also observed decreased hepatic expression of total IRS1 in both DHT-treated groups, as this inhibitory phosphorylation triggers degradation of IRS1 by ubiquitination (28).

There are several possible mechanisms leading to impaired insulin signaling in the liver, including increased FFA flux, lipid accumulation, and inflammation *in situ* (29). In our previous research, we did not find increased FFA in the circulation (18) or lipid accumulation in the liver (under review), ruling out these mechanisms as likely causes of insulin resistance in the liver. Furthermore, one of the most important mechanisms contributing to reduced insulin sensitivity, at both systemic and tissue levels, is inflammation, which together with insulin resistance forms a vicious cycle in which each condition promotes the other (30). We examined inflammation in the liver at the level of proinflammatory transcription factors JNK and NFκB, as well as their upstream regulators and downstream mediators. We showed that postnatal overfeeding is the dominant factor leading to increased activation of JNK1, as estimated by an increased ratio of pJNK^Thr183/Tyr185^/JNK1. This is associated with an increased level of NLRP3 inflammasome complex and IL1β mRNA only in small litter raised DHT-treated animals, as a result of the interaction of factors. These results are consistent with previous studies reporting increased NLRP3 and IL1β levels in the PCOS patients (31). The relationship between NLRP3 and JNK in the liver of PCOS patients is not directly addressed in the literature, and these findings suggest their possible interactions in the context of PCOS. On the other hand, the expression of TLR4 and TNFα was only affected by hyperandrogenemia. The link between these proinflammatory mediators could be NFκB (32), but in our study NFκB was not activated. One of the key mediators of the activated NFκB signaling in the ovaries of PCOS rats is MIF, as it has been shown that MIF can promote NFκB expression in the ovarian cytoplasm (33). In this study, both treatments decreased hepatic MIF mRNA levels, which could be one of the possible reasons for the attenuated NFκB signaling.

It is known that activation of inflammatory pathways via serine kinase phosphorylation of IRS1 and IRS2 establishes a link between inflammation and insulin resistance. We hypothesize that the trigger for the observed impairment of insulin signaling is the inhibitory phosphorylation of IRS-1 on Ser307 promoted by JNK, as demonstrated by Nakatani et al. (5).

Importantly, the induction of inflammation together with systemic insulin resistance and impaired insulin signaling in the liver was most evident when both postnatal overfeeding and hyperandrogenemia were present. Early postnatal overfeeding in rodents, triggered by litter size reduction, is a model that according to the literature corresponds to the development of prepubertal obesity in humans and increased susceptibility to metabolic disease (34). The small litter model used in this study did not lead to the obesity in animals, but to overweight and visceral adiposity, which mimics early prepubertal overweight. These metabolic features were not followed with steatosis, lobular inflammation or ballooning characteristic for MASLD (manuscript under review). The prepubertal period is critical for the development of PCOS (35), and our study has shown that increased adiposity in this period favors the development of PCOS-associated inflammation and insulin resistance. This is in accordance with a study of letrozole-induced PCOS animal model, in which it was shown that prepubertal high-fat diet induced both metabolic and ovarian disturbances similar to those observed clinically in women with PCOS (36).

An important factor that contributes to the regulation of insulin sensitivity in the liver is the energy sensor AMPK. Dysregulation of AMPK and SIRT1 correlates with insulin resistance as well as with other endocrine and reproductive features of PCOS (37). In our study, the pAMPK^Thr172^/AMPK ratio, which indicated the degree of AMPK activation, was decreased in small litter raised DHT-treated animals, due to interaction of the factors. This led to lower AMPK activity and thus to reduced inhibitory Ser^79^ phosphorylation of its target protein ACC, which directs fatty acids towards lipogenesis at the expense of beta-oxidation (38). Since SIRT1 is the main target in AMPK regulation of energy metabolism (12), in accordance with the reduced AMPK activity, the nuclear protein level of SIRT1 was also reduced in small litter raised DHT-treated group. SIRT1 was shown to modulate the activity of LKB1, an important AMPK kinase, suggesting a reciprocal regulatory relationship between SIRT1 and AMPK (39). In agreement with our results, dysregulation of the AMPK-SIRT1 signaling pathway was found in ovarian tissue of other PCOS rat models (40, 41). Therefore, it has been suggested that the protective effect of AMPK on insulin resistance in PCOS rats is mediated by the AMPKα-SIRT1 signaling pathway (40). This is consistent with our previous results showing that AMPK activation is critical for the maintenance of insulin sensitivity in VAT in this PCOS animal model (18).

Our previous results have shown that overfeeding in the early postnatal period together with DHT treatment leads to hyperinsulinemia and systemic insulin resistance in the DHT-induced model of PCOS, while the results of this study show that this is associated with increased inflammation and impaired regulation of energy metabolism in the liver. While postnatal overfeeding tends to stimulate hepatic JNK1 activation, the presence of both factors triggers activation of NLRP3 and increased expression of IL1β. This proinflammatory state is accompanied by decreased phosphorylation of AMPK and reduced activity or levels of its target proteins (inhibitory ACC^Ser79^ phosphorylation and SIRT1) and impaired hepatic insulin signaling. On the other hand, the increased expression of TLR4 and TNFα is exclusively associated with the effect of DHT, although mechanism remains to be clarified. Overall, these results indicate that increased food intake in postnatal period promotes the development of insulin resistance in the liver of the DHT-induced PCOS animal model, and suggest that hepatic inflammation and dysregulation of energy metabolism may be involved in the disruption of insulin signaling in this tissue. According to these results, activation of AMPK and/or inhibition of NLRP3 activation could provide a basis for the development of new therapeutic strategies for the treatment of insulin resistance in women with PCOS.

## Data Availability

The raw data supporting the conclusions of this article will be made available by the authors, without undue reservation.
